# Clinical and Nonclinical Effects on Operative Duration: Evidence from a Database on Thoracic Surgery

**DOI:** 10.1155/2020/3582796

**Published:** 2020-02-10

**Authors:** Jin Wang, Javier Cabrera, Kwok-Leung Tsui, Hainan Guo, Monique Bakker, John B. Kostis

**Affiliations:** ^1^Institute of Physical Internet, School of Intelligent Systems Science and Engineering, Jinan University (Zhuhai Campus), Zhuhai, China; ^2^School of Data Science, Department of Systems Engineering and Engineering Management, City University of Hong Kong, Hong Kong, China; ^3^Department of Statistics and Biostatistics, Rutgers University, Piscataway, NJ 08854, USA; ^4^Research Institute of Business Analytics and Supply Chain Management, College of Management, Shenzhen University, Shenzhen 518060, China; ^5^Cardiovascular Institute, Rutgers Robert Wood Johnson Medical School, New Brunswick, NJ 08904, USA

## Abstract

**Background:**

Due to the high maintenance costs, it is critical to make full use of operating rooms (ORs). Operative duration is an important factor that guides research on surgery scheduling. Clinical effects, for example, surgery type, rationally influences operative duration. In this study, we also investigate whether the planning and scheduling decisions in ORs influence the operative duration.

**Methods:**

For our study, we collected and reviewed data on 2,451 thoracic operations from a large hospital in China. The study was conducted over a period of 34 months. Linear and nonlinear regression models were used to detect the effects on the duration of the operations. We have also examined interactions between the factors.

**Results:**

Operative duration decreased with the number of operations a surgeon performed in a day (*P* < 0.001). It was also found that operative duration decreased with the number of operations allocated to an OR, as long as there were not more than four surgeries per day (*P* < 0.001). It was also found that operative duration decreased with the number of operations allocated to an OR, as long as there were not more than four surgeries per day (*P* < 0.001). It was also found that operative duration decreased with the number of operations allocated to an OR, as long as there were not more than four surgeries per day (

**Conclusions:**

Operative duration was affected not only due to clinical effects but also some nonclinical effects. Scheduling decisions significantly influenced operative duration.

## 1. Introduction

It is important to utilize the operating rooms (ORs) well since the OR costs account for a great part of the expenses in hospitals. OR costs are largely based on the planning and scheduling decisions, which are made according to the information related to the duration of operations. Therefore, it is important to investigate which factors influence operating durations. Usually, it is believed that operative duration depends on factors related to clinical issues or patients [1], e.g., age, gender, and body mass index. It is also found that operative duration is affected by the surgeon's operating skills [2, 3], assistant surgeons [4, 5] management policies [6], switching of teams [7], and surgeon fatigue [8]. Predictors of operative duration of general thoracic surgeries are identified in [9]. However, nonclinical factors have not been taken into consideration for understanding the effects on operative durations to the same extent. Nevertheless, researchers have examined the effects of nonclinical factors such as length of stay (LOS), hospital's history of mortality rate, and readmission rate. The typical nonclinical factors include the day of the week, occupancy rate in the medical ward, and the physician's workload. It is shown that admission on weekends results in higher mortality [10, 11], and higher workload results in shorter LOS [12, 13], high number of readmissions [13], and high mortality rates [14]. As for the workload in ORs, previous investigators find that high workload is associated with work distractions [15]. It is worth noting that the workload in ORs is different from that in medical wards. This can be explained as the workload in medical wards is often represented by the number of patients in a given clinical unit since the work is performed nearly simultaneously. In contrast, in the ORs, the surgeries are scheduled in a regular order, and the operations of a surgeon are performed sequentially. It would be interesting to investigate the effects of the workload consisting of sequential jobs. Therefore, this paper is motivated by the studies considering clinical effects on operative duration as well as those relevant to nonclinical effects on medical services.

The aim of this study is to identify the factors that influence operative duration from both clinical and nonclinical perspectives. Specifically, this paper focuses on the clinical factors: (1) surgery types and surgeons performing the procedure, and the nonclinical factors (2) days of the week, (3) workloads (i.e., the surgeon's workload the workload in the OR), and (4) the sequencing order of surgeries performed. Hence, we hypothesize as follows.

**Hypothesis 1 .**

Operative duration depends on the type of surgery and on the surgeon performing the surgery.

Past literature focused on if and how surgeries performed during weekends affected operative duration [10, 11, 16]. In addition, the day of a week might also influence operative duration since aside from operations in the ORs, and surgeons may have other work responsibilities in different weekdays, for example, administrative jobs and attending outpatients, which will have already been planned several weeks ago. These additional job responsibilities may also influence operative durations. We thus hypothesize as follows.

**Hypothesis 2 .**

The day of the week has an effect on operative duration.

Based on the assumption that the workload has an effect on other situations, we hypothesize the following hypothesis.

**Hypothesis 3 .**

The operative duration decreases with the amount of workload, i.e., (a) the number of surgeries performed by the surgeon in a day and (b) the number of surgeries scheduled in the OR.

Additionally, we hypothesize the following.

**Hypothesis 4 .**

The time of the day has an influence on the duration of surgery, if comparing same procedures.

The contributions of this paper include as follows: (1) we examine the factors that influence operative durations from both clinical and nonclinical perspectives, (2) we find the nonlinear relationship between workloads in ORs and surgery duration, and (3) we find the order of surgeries a surgeon performs in a day impacts operative duration.

## 2. Materials and Methods

### 2.1. Data Source

After the approval of the institutional review board, data were collected from the department of thoracic surgery from the Hospital Affiliated with Dalian Medical University, Liaoning province, China. It is one of the largest hospitals in the province and is one of the top 100 hospitals in China. The department of thoracic surgery is the largest department in the hospital. Data were collected over the period of January 2014 through October 2016 and included 2,451 observations. All operations included in this report are thoracic surgeries, of which pulmonary lobectomy accounted for more than half (67.39%) the operations.

Compared with the ACS-NSQIP database that was often quoted in the literature (e.g., [1, 4]), our database included some additional variables, i.e., surgeon-specific variable, OR-specific variable, and the variable on the detailed timeline and sequencing of each surgery. Specifically, our data included the information about (1) the surgeon and the anaesthetist of each surgery, (2) the OR where the surgery was performed, and (3) detailed time taken for each surgery (i.e., (i) the time a patient was wheeled in, into the OR, (ii) the time when the patient was given anaesthesia, (iii) the time when the surgeon began to cut, (iv) the time when the surgeon finished sewing, and (v) the time when the patient was wheeled out of the OR). This allowed the analysis of the duration of surgery with regard to information related to the surgeon and the OR (time taken for the surgery in the OR). The sample data with three records are illustrated in Table 1. Note that the personal information related to the surgeons is kept anonymous throughout the paper to protect their privacy.

### 2.2. Study Variables

The primary variables included in our study are the surgeon performing the procedure, the number of surgeries a surgeon performed in a day, the orderings (the position of the surgery in the sequence of surgeries performed by the surgeon on that day), the number of surgeries scheduled in the OR where the surgery was performed, and the day of the week when the surgery was performed. Other variables included the surgery type and the anaesthetist. The Hospital Affiliated with Dalian Medical University hospital did not use the ICD-10 code. The hospital categorized surgeries into the specific surgery type, according to an internal manual of the hospital. Our database consists of 11 surgery types.

### 2.3. Outcome Variables

Normally, the medical staff involved in an operation includes nurses, anaesthetists, a surgeon, and assistants. The surgery procedure is divided into four parts according to the five time points mentioned above. The first two parts and the last part are mainly completed by nurses and anaesthetists. The third part is mainly performed by the surgeon and his/her assistants since this part is critical for the quality/success of the surgery, and surgeons are the critical human resource the hospital and are held responsible for the success/failure of the surgery. We focused on the effects of the surgeons, and the primary outcome in this paper is the length of the third part of the operations, i.e., the cut-suture time. The log of operative duration is used as the outcome variable in order to avoid the skewed distributions.

### 2.4. Statistical Analysis

**Figure 1:**
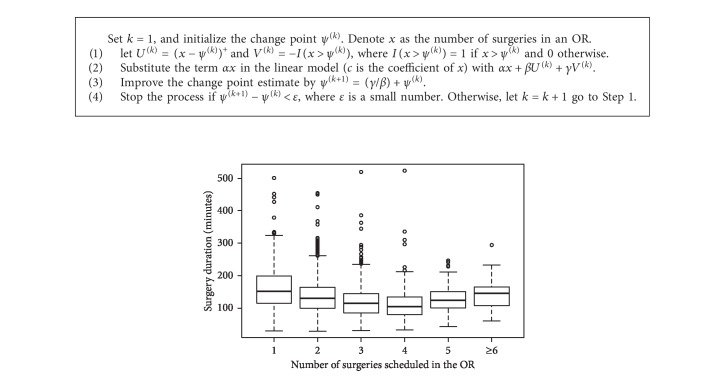
The box-plot about the relationship between operative duration and the number of surgeries in an OR. In the cases that there are four or fewer surgeries in an OR, the surgery duration decreases with the number of surgeries, while the duration increases if the number of surgeries in an OR is five or more.

We first examined the effects of the primary variables. Since the two variables, i.e., the number of surgeries a surgeon performed in a day and the surgery position in the scheduling sequence, are collinear. Two linear regression models (model I and model II) with either of the variables are used to examine the effects. The relationship between variables should be considered [17, 18], and hence this study incorporated not only linear but also nonlinear models. The box-plot of the operative duration in two different ranges (cut-off on 4) of the number of surgeries in an OR (Figure 1) showed a change point. Specifically, when there are 4 or fewer surgeries allocated in an OR, the operative duration of the surgeries decreases, whereas the duration increases if there are more than 4 surgeries in the OR. This suggests the existence of a change point. We applied the algorithm proposed in [19] to test it. The pseudocode of the algorithm is as follows (Algorithm 1).

We performed variable selection by applying the LASSO method. The sample size of 2451 is quite sizeable, so we did not perform a power analysis. The technical details are available in [20]. Also, a preliminary version of this paper was posted on [21].

## 3. Results and Discussion

### 3.1. Results

We present the regression results in Table 2, and use the referral of 0.005 as a threshold for statistical significance. We interpret the results for the hypotheses as follows.

Hypothesis 1 was supported by the models. The regression results in Table 2 found that operative duration greatly depended on the surgery type, as based on the complexity involved in the procedure. The results illustrated the differences in operative duration for different surgery types. The baseline was the surgery with the most number of instances, named the thoracoscopic interior pulmonary lobectomy.

The regression results in Table 2 illustrated that the mean of operative duration was relevant to the surgeon performing the procedure. Surgeons B and F were significant, whose coefficients are −0.094 and −0.272. That is, the mean of the duration of surgeries performed by surgeons B (*P* < 0.001) and F (*P* < 0.001) was 117 minutes (95% confidence interval [114, 121]) and 110 minutes (95% confidence interval [92, 109]), about 13 minutes and 36 minutes less than that performed by surgeon A (the intercept is 4.97).

Hypothesis 2 was not supported by the two models. Significant effects of the day of the week were observed (Table 2). The days were all not significant. Tuesday and Friday had the small *P* values, but still larger than 0.01.

**Figure 2:**
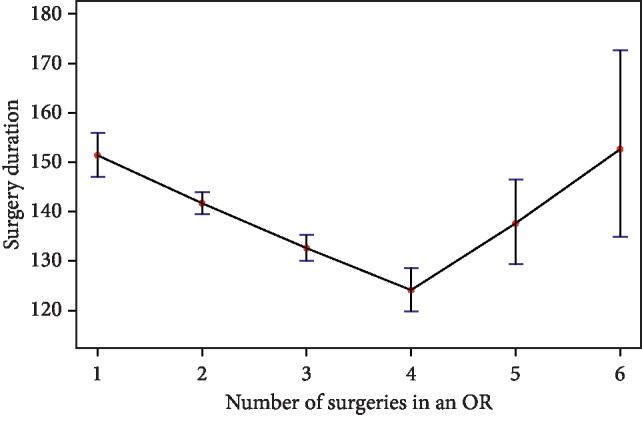
The relationship between the operative duration and the number of surgeries in an OR in a day. When there were no more than four surgeries in an OR, operative duration decreased around 8 minutes if one more surgery was allocated into the OR; when there were more than four surgeries in the OR, operative duration increased around 12 minutes for one more surgery additional surgery into the OR. The confidence intervals of 5 and 6 are much larger than others.

Hypothesis 3(a) was supported by model I. The regression results of model I showed that operative duration decreased with the number of surgeries a surgeon performed in a day since the coefficient is −0.061. With the use of the algorithm in [19], the change point (as a continuous variable) converged quickly to 3.94, which was very close to the observed change point, 4. Hence, we formulated the piece-wise linear regression models. Hypothesis 3(b) was rejected by the two models. The results of models I and II demonstrated that a change point of the workload in OR existed. We plotted the mean and confidence interval in Figure 2. When there were no more than four surgeries in an OR, operative duration decreased if one more surgery was scheduled in the OR (the coefficient is −0.067); when there were more than four surgeries in the OR, operative duration for one additional surgery in the OR (the coefficient is 0.109). Additionally, the confidence intervals of 5 and 6 ([128, 137] and [144, 164], respectively) were much larger than others.

Hypothesis 4 was tested by model II. As we mentioned before, the schedule for operations for a surgeon was prepared in a sequential order. Hence, the workload pressure for surgeons is high for surgeries early in the day, not in the later part of the day. The regression results of model II verified this conjecture. However, this is not significant when a surgeon performed five surgeries in a day, they only handled 1.18% of the cases. The coefficients were all negative, indicating that the mean of operative duration decreased if a surgeon performed more than one surgery in a day. Also, the mean of the duration of the first surgery was less compared with the later surgeries, when a surgeon performed two, three, or four surgeries in a day. For example, the coefficient of 4∼1 was −0.206, which meant that when a surgeon performed four surgeries in a day, the mean of the duration of the first surgery was 117 minutes (95% confidence interval [104, 130]), a decrease by about 26 minutes (*P* < 0.001).

### 3.2. Discussion

The study had significant strengths including the large numbers of operations, the uniformity of the administrative procedures in the hospital, which benefited the analysis of the effects of clinical and nonclinical factors. We found surgeons tended to accelerate their work when performing more number of surgeries. The most interesting finding in this study was that the workloads in an OR influence the operative duration. The increase of more than 4 surgeries in an OR might be because when too many surgeries were allocated to an OR, it would become disordered, which would result in longer operative duration. This study was insightful for surgery-scheduling problems since the basic assumption that scheduling decisions were independent of surgery duration was denied. With this new finding, new models should be formulated.

In the literature, the interactions between factors were considered to affect the OR efficiency and patient safety, such as pairing surgeons with anaesthesiologists [22, 23] and pairing surgeons and the assistants [4]. However, we tested these two kinds of interactions, but no items were significant. We also investigated other interactions, i.e., the interactions among surgeons, surgery types, and ordering sequences. The coefficients of surgeons and surgery positions in the sequencing, and their interactions are shown in Table 3. Surgeons B and C were flexible; the duration of these two surgeons' surgeries depended significantly on the surgery positioning. Specifically, surgeon B was much faster when they performed the surgeries in position 4∼3 (*P* < 0.001).

### 3.3. Limitations of the Study

The principal limitation of this study is that the results are obtained based only on one database of a department of one hospital. The surgery types were also limited, which are mainly thoracic surgeries. Hence, more data should be used to verify whether the results in this paper hold for other surgeons and surgery types. In addition, if more data are available in future, the effects of other factors might be investigated, such as turnover time [24], interruptions [25–27], and surgeon experience [28, 29], since these factors have been found to affect the surgical quality.

Additionally, the fact that a surgeon working faster (or slower) does not mean that he/she is better (or worse) at the surgeries. Hence, another limitation is that the outcomes of the surgeries (e.g., length of stay, readmission rate, and mortality rate) are not mentioned in this paper. The effect of surgery duration on healthcare quality is worthy of attention. We should take both efficient planning and surgical quality into account seriously.

## 4. Conclusions

In this paper, the clinical and nonclinical effects on operative duration were investigated based on a dataset on thoracic surgeries. Instead of only focusing on the clinical factors, nonclinical factors were also studied. It was found that the operative duration was influenced by surgeons' workload and workload in the OR where the surgery was performed. The duration decreased with surgeons' workload. However, it did not monotonically decrease with the workload in the OR. Specifically, the operative duration decreased with the number of surgeries in the OR in a day if it was not more than four, whereas it would increase with the number if it was beyond four. Also, the duration was impacted by the position of the surgery in a sequence of surgeries a surgeon performed in a day. In addition, the interactions among surgeons, surgery types, and surgery positions also influenced operative duration.

## Figures and Tables

**Algorithm 1:**
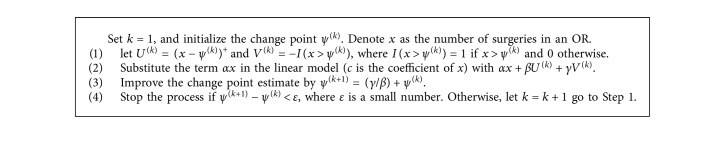
LFFSA.

**Table 1:** The sample data with three records.

ID	Sex	Age	Date	Day	OR	Surgeon
6952 × 5	F	46	6/1/2016	Thu	OR10	A
69 × 504	F	29	7/1/2016	Fri	OR02	A
69 × 541	M	57	8/1/2016	Sat	OR15	A

Surgery type	Anaesthetist	Enter time	Anaesthesia time	Cut time	Sew time	Leave time

Surgery 11	A	11 : 15	11 : 29	12 : 18	13 : 25	13 : 40
Surgery 4	B	13 : 06	13 : 21	13 : 43	15 : 20	15 : 30
Surgery 4	C	10 : 54	11 : 56	12 : 28	14 : 00	14 : 10

**Table 2:** Regression results for models using only the main effects and without interaction terms. Anaesthetist was excluded because of space.

Predictors	Percentage (%)	Results of model I			Results of model II		
		Coefficient	95% confidence interval	*P* value	Coefficient	95% confidence interval	*P* value
The day of the week^†^							
Sunday	1.22	−0.041	(−0.188, 0.106)	0.582^$^	−0.045	(−0.193, 0.104)	0.556^$^
Monday	1.80	−0.065	(−0.188, 0.058)	0.298^$^	−0.077	(−0.201, 0.048)	0.227^$^
Tuesday	20.77	0.056	(0.004, 0.109)	0.036^*∗*^	0.057	(0.005, 0.11)	0.033^#^
Wednesday	20.03	0.033	(−0.02, 0.086)	0.224^$^	0.035	(−0.018, 0.088)	0.197^$^
Thursday	17.75	0.007	(−0.047, 0.062)	0.790^$^	0.009	(−0.046, 0.063)	0.755^$^
Friday	22.97	0.054	(0.003, 0.106)	0.039^*∗*^	0.053	(0.002, 0.105)	0.042^#^
Number of surgeries in the OR in a day^#^							
Less than or equal to 4		−0.066	(−0.089, −0.043)	<0.001^*∗∗∗*^	−0.067	(−0.09, −0.044)	<0.001^*∗∗∗*^
More than 4		0.103	(0.034, 0.172)	0.003^*∗∗*^	0.109	(0.04, 0.178)	0.002^*∗∗*^
Number of surgeries a surgeon performed in a day^#^		−0.061	(−0.08, −0.043)	<0.001^*∗∗∗*^			
Position^‡^							
2 surgeries in a day	39.86						
2∼1					−0.075	(−0.123, −0.027)	0.002^*∗∗*^
2∼2					−0.067	(−0.115, −0.019)	0.006^*∗∗*^
3 surgeries in a day	24.97						
3∼1					−0.169	(−0.234, −0.104)	<0.001^*∗∗∗*^
3∼2					−0.145	(−0.209, −0.081)	<0.001^*∗∗∗*^
3∼3					−0.163	(−0.228, −0.098)	<0.001^*∗∗∗*^
4 surgeries in a day	9.06						
4∼1					−0.206	(−0.317, −0.095)	<0.001^*∗∗∗*^
4∼2					−0.141	(−0.257, −0.026)	0.017^*∗*^
4∼3					−0.129	(−0.238, −0.02)	0.021^*∗*^
4∼4					−0.148	(−0.255, −0.042)	0.006^*∗∗*^
5 surgeries in a day	1.18						
5∼1					−0.287	(−0.601, 0.027)	0.073^&^
5∼2					−0.051	(−0.364, 0.262)	0.749^$^
5∼3					−0.132	(−0.445, 0.182)	0.410^$^
5∼4					−0.443	(−0.784, −0.102)	0.011^*∗*^
5∼5					−0.105	(−0.419, 0.208)	0.510^$^
Surgeons^§^							
Surgeon B	20.69	−0.094	(−0.139, −0.048)	<0.001^*∗∗∗*^	−0.098	(−0.144, −0.052)	<0.001^*∗∗∗*^
Surgeon C	19.75	0.058	(0.015, 0.107)	0.011^*∗*^	0.059	(0.013, 0.104)	0.011^#^
Surgeon D	12.08	−0.006	(−0.058, 0.042)	0.826^$^	−0.008	(−0.061, 0.045)	0.767^$^
Surgeon E	6.94	−0.034	(−0.110, 0.032)	0.329^$^	−0.039	(−0.109, 0.03)	0.271^$^
Surgeon F	3.06	−0.272	(−0.366, 0.178)	<0.001^*∗∗∗*^	−0.270	(−0.365, −0.176)	<0.001^*∗∗∗*^
Surgery type^Δ^							
1. Lung cancer	4.04	0.155	(0.073, 0.237)	<0.001^*∗∗∗*^	0.157	(0.075, 0.239)	<0.001^*∗∗∗*^
2. Thoracoscopic pulmonary bullous resection	2.49	−0.369	(−0.476, −0.263)	<0.001^*∗∗∗*^	−0.375	(−0.482, −0.267)	<0.001^*∗∗∗*^
3. Thoracoscopic partial pulmonary lobectomy	18.07	0.009	(−0.037, 0.054)	0.710^$^	0.009	(−0.036, 0.055)	0.684^$^
4. Total pneumonectomy	1.10	0.388	(0.238, 0.538)	<0.001^*∗∗∗*^	0.391	(0.241, 0.541)	<0.001^*∗∗∗*^
5. Partial pulmonary lobectomy	11.42	0.110	(0.056, 0.163)	<0.001^*∗∗∗*^	0.108	(0.054, 0.161)	<0.001^*∗∗∗*^
6. Thoracoscopic exploration	1.31	0.031	(−0.106, 0.168)	0.655^$^	0.033	(−0.104, 0.169)	0.641^$^
7. Pulmonary wedge resection	2.37	−0.077	(−0.182, 0.027)	0.145^$^	−0.077	(−0.181, 0.027)	0.147^$^
8. Esophageal cancer	2.04	0.774	(0.661, 0.887)	<0.001^*∗∗∗*^	0.773	(0.660, 0.885)	<0.001^*∗∗∗*^
9. Mediastinal tumor resection	5.79	−0.170	(−0.239, −0.101)	<0.001^*∗∗∗*^	−0.168	(−0.237, −0.099)	<0.001^*∗∗∗*^
10. Pulmonary tumor resection	13.42	−0.043	(−0.093, 0.007)	0.091^&^	−0.043	(−0.093, 0.007)	0.089^&^
Adjusted *R*^2^		0.198			0.203		

Significant codes: 0 “^*∗∗∗*^”; 0.001 “^*∗∗*^”; 0.01 “^*∗*^”; 0.05 “^&^”; and 0.1 “^$^”. ^†^Categorical variable, the baseline is Saturday. ^#^Continuous variables. ^‡^Categorical variable; the baseline is the fact that a surgery is the only one surgery a surgeon performed in a day. Notation “2∼1” means the first surgery in the day with two surgeries performed by a surgeon in the day. ^§^Categorical variable; the baseline is surgeon A who performed most surgeries. ^Δ^Categorical variable; the baseline is a surgery type, named thoracoscopic interior pulmonary lobectomy.

**Table 3:** The regression results relevant to surgeons, surgery positions, and their interaction. The coefficients of other variables were not listed in the table since they were quite similar to those of model II.

Variables	Coefficient	95% confidential interval	*Pr*(>|*t*|)
Surgeon			
Surgeon C	0.074	[0.031, 0.118]	0.001^*∗∗∗*^
Surgeon F	−0.269	[−0.359, −0.180]	<0.001^*∗∗∗*^

Surgery position			
Position 3∼1	−0.155	[−0.217, −0.092]	<0.001^*∗∗∗*^
Position 3∼3	−0.085	[−0.146, −0.024]	0.007^*∗∗*^
Position 4∼1	−0.165	[−0.268, −0.061]	0.002^*∗∗*^
Position 4∼2	−0.102	[−0.211, 0.007]	0.067^&^
Position 4∼4	−0.100	[−0.200, −0.001]	0.048^*∗*^
Position 5∼1	−0.422	[−0.755, -0.089]	0.013^*∗*^

Interactions			
Surgeon B: position 2∼1	−0.141	[−0.218, −0.064]	<0.001^*∗∗∗*^
Surgeon B: position 2∼2	−0.141	[−0.221, −0.061]	0.001^*∗∗∗*^
Surgeon B: position 3∼2	−0.228	[−0.366, −0.091]	0.001^*∗∗*^
Surgeon B: position 3∼3	−0.171	[−0.319, −0.024]	0.023^*∗*^
Surgeon B: position 4∼3	−0.434	[−0.642, −0.225]	<0.001^*∗∗∗*^
Surgeon B: position 5∼5	−0.393	[−0.917, 0.131]	0.141^$^
Surgeon C: position 3∼1	0.150	[0.010, 0.290]	0.036^*∗*^
Surgeon C: position 3∼2	−0.258	[−0.393, −0.123]	<0.001^*∗∗∗*^
Surgeon C: position 5∼4	−0.756	[−1.283, −0.229]	0.005^*∗∗*^
Surgeon D: position 5∼1	0.980	[0.169, 1.790]	0.018^*∗*^
Surgeon E: position 3∼2	−0.361	[−0.790, 0.068]	0.099^&^
Surgeon E: position 3∼3	−0.428	[−0.766, −0.089]	0.013^*∗*^
Adjusted *R*^2^	0.222		

## Data Availability

The dataset used and/or analyzed during the current study is not publicly available because it contains very sensitive and private information about patients and surgeon. But the dataset is available from the corresponding author on reasonable request.
